# Supplementary material for: Structural basis for transcription antitermination at bacterial intrinsic terminator
Source: Nat Commun. 2019 Jul 11;10:3048. doi: 10.1038/s41467-019-10955-x (PMC6624301; doi:10.1038/s41467-019-10955-x)
Supplement: Supplementary file 1 — Supplementary Information [file 41467_2019_10955_MOESM1_ESM.pdf]

Structural basis for transcription antitermination at bacterial intrinsic terminator

Linlin You *et al.*

Supplementary Table 1. The statistics of cryo-EM structures of *Xoo* P7-TEC and P7-NusA-TEC

	***Xoo* P7-TEC**	***Xoo* P7-NusA-TEC**
**Data collection**		
Number of grids used	1	1
Grid type	Holey carbon	Holey carbon
Microscope/detector	Titan Krios/Gatan K2	Titan Krios/Gatan K2
Voltage (keV)	300	300
Dose rate (e^-/s)	6.7	6.24
Pixel size (Å/pix)	0.507	0.507
Total dose (e^-/ $Å^2$)	53.6	49.92
Total exposure time (s)	8	8
Number of frames/movie	32	32
Defocus range (μm)	2.0-2.6	2.0-2.5
Number of micrographs	2271	3702
Particles used for final map	319,613	204,450
**Model composition**		
Non-hydrogen atoms	23658	24427
Protein residues	3059	3203
Nucleotide	63	63
Ligands (Zn2+/Mg2+)	2/1	2/1
**Refinement**		
Resolution (Å)	3.95	3.41
Map sharpening B factors ($Å^2$)	-90.575	-153.75
Clash score	3.52	2.56
Average B factor ($Å^2$)		
Protein	70.46	69.5
Nucleotide	119.22	128.03
Ligand	75.26	76.79
RMS deviations		
Bond lengths (Å)	0.003	0.003
Bond angles (°)	0.611	0.623
Ramachandran plot		
Favored (%)	96.78	96.04
Allowed (%)	3.22	2.96
Outliers (%)	0	0

Supplementary Table 2. Plasmids used in this study

Reagent or Resource	Source	Identifier
pET-28a-TEV	Gift from Zhaocai Zhou's lab	N/A
pET-28a-TEV-P7	This study	N/A
pET-28a-TEV-P7(D43A)	This study	N/A
pET-28a-TEV-P7(Y44A)	This study	N/A
pET-28a-TEV-P7(S46W)	This study	N/A
pET-28a-TEV-P7(P48W)	This study	N/A
pET-28a-TEV-P7(E49W)	This study	N/A
pET-28a-TEV-P7(F50A)	This study	N/A
pET-28a-TEV-P7(V51A)	This study	N/A
pET-28a-TEV-P7(A52E)	This study	N/A
pET-28a-TEV-P7(E53W)	This study	N/A
pET-28a-TEV-P7(R73C)	This study	N/A
pET-28a-TEV-P7(ΔCTL; Δ69-73)	This study	N/A
pET-28a-*Xoo* σ^{70}	This study	N/A
pTolo-EX5-*Xoo NusA*	This study	N/A
pACYC-*Xoo rpoA*-*rpoZ*	This study	N/A
pCOLA-*Xoo rpoB*-*rpoC*	This study	N/A
pCOLA-*Xoo rpoB*-*rpoC* (Δβ'NTH; Δ1-10)	This study	N/A
pARTaq-N25-100-tR2	Gift from Richard Ebright's lab	N/A
pARTaq-N25-*his*-tR2	This study	N/A
pARTaq-N25(Ext-10)	This study	N/A
pARTaq-N25(Anti-35+Ext-10)	This study	N/A

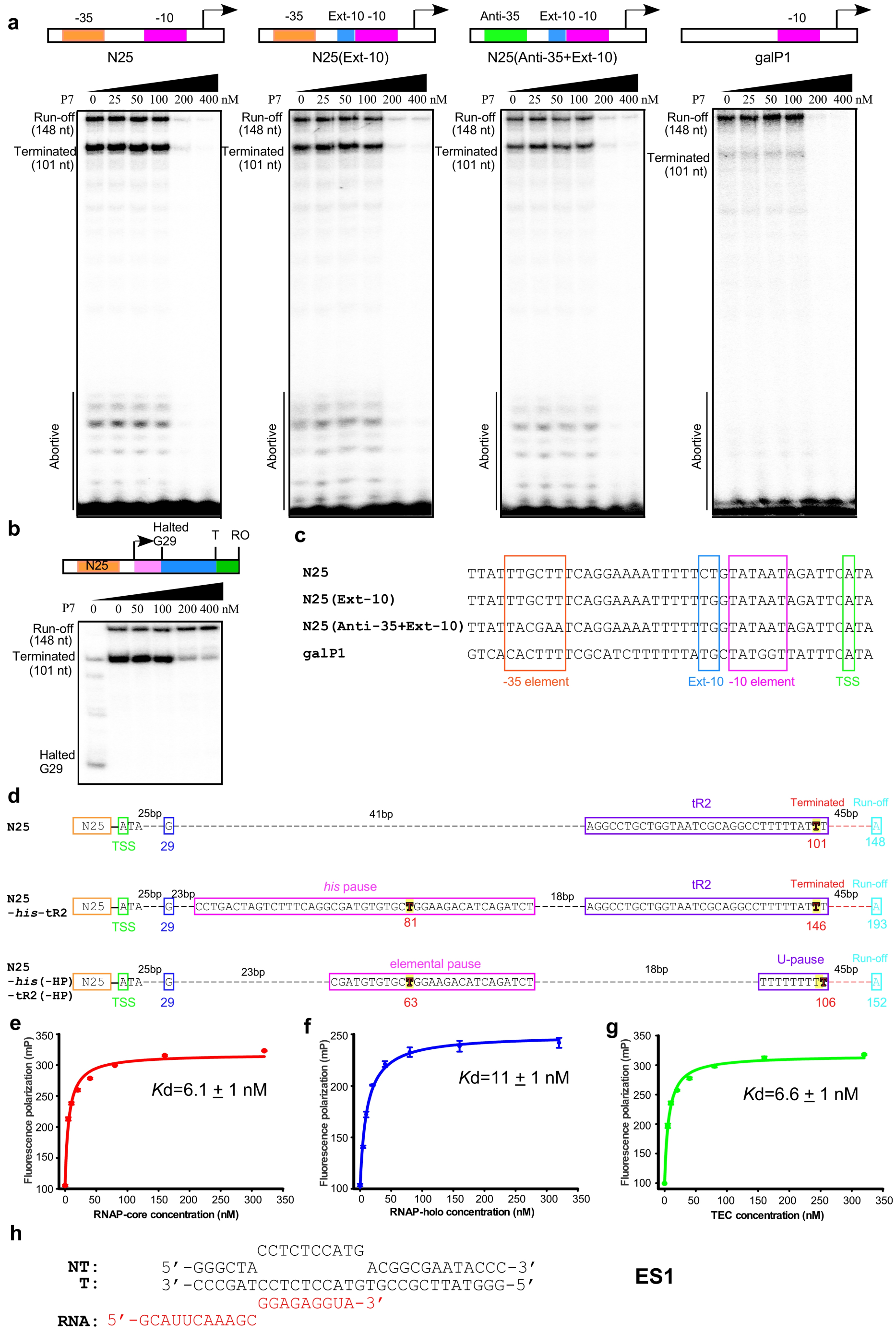

Supplementary Figure 1. P7 inhibits transcription initiation and termination. Related to Figure 1. (a) P7 inhibits transcription initiation from N25, N25 derivatives and galP1 promoters. **(b)** P7 prevents transcription termination at the tR2 terminator. The first lane represents RNA transcripts halted at +29. **(c)** The promoter sequences used in the (a). **(d)** The sequences of promoter template used in (b) (N25), in Fig. 3h (N25-*his*-tR2), and in Fig. 3i (N25-*his*(-HP)-tR2-(-HP)). **(e)** P7 binds to RNAP core enzyme with a *K*d value of 6.1 nM in a FP assay using a fluorescein-labeled P7. **(f)** P7 binds to RNAP holoenzyme with a *K*d value of 11 nM in a FP assay using a fluorescein-labeled P7. **(g)** P7 binds to RNAP TEC with a *K*d value of 6.6 nM in a FP assay using a fluorescein-labeled P7. The experiments were repeated in triplicate, and the data were presented as mean ± S.E.M. **(h)** The elongation nucleic-acid scaffold (ES1) used in (**g**). The source data of supplementary Fig. 1e-g are provided in the Source Data file.

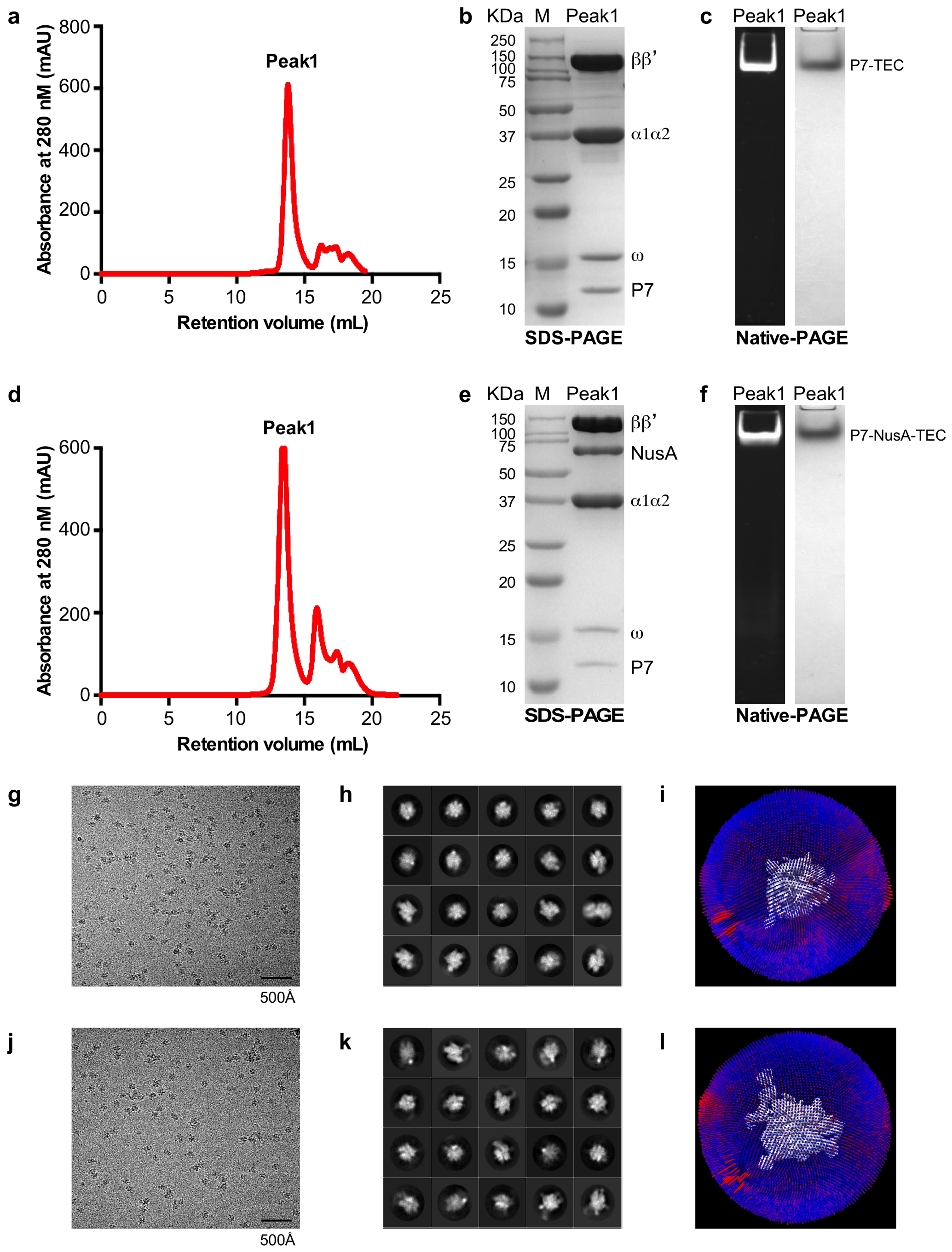

Supplementary Figure 2. The complex reconstitution of P7-TEC and P7-TEC-NusA. Related to Figure 1. (a) Elution peaks of *Xoo* P7-TEC from a size-exclusion column. Peak 1 is the *Xoo* P7-TEC. **(b)** The SDS-PAGE and **(c)** the native-PAGE of peak 1. The gel was first stained with SYBR Gold for nucleic acids and then with Coomassie Brilliant Blue for proteins. **(d)** Elution peaks of *Xoo* P7-NusA-TEC from a size-exclusion column. Peak 1 is the *Xoo* P7-NusA-TEC. **(e)** The SDS-PAGE and **(f)** the native-PAGE of peak 1 in (d). **(g)** The representative cryo-EM micrograph of *Xoo* P7-TEC. **(h)** The representative 2D classifications of *Xoo* P7-TEC single particles. **(i)** The angular distribution of *Xoo* P7-TEC particle projections. **(j)** The representative cryo-EM micrograph of *Xoo* P7-NusA-TEC. **(k)** The representative 2D classifications of *Xoo* P7-NusA-TEC single particles. **(l)** The angular distribution of *Xoo* P7-NusA-TEC particle projections.

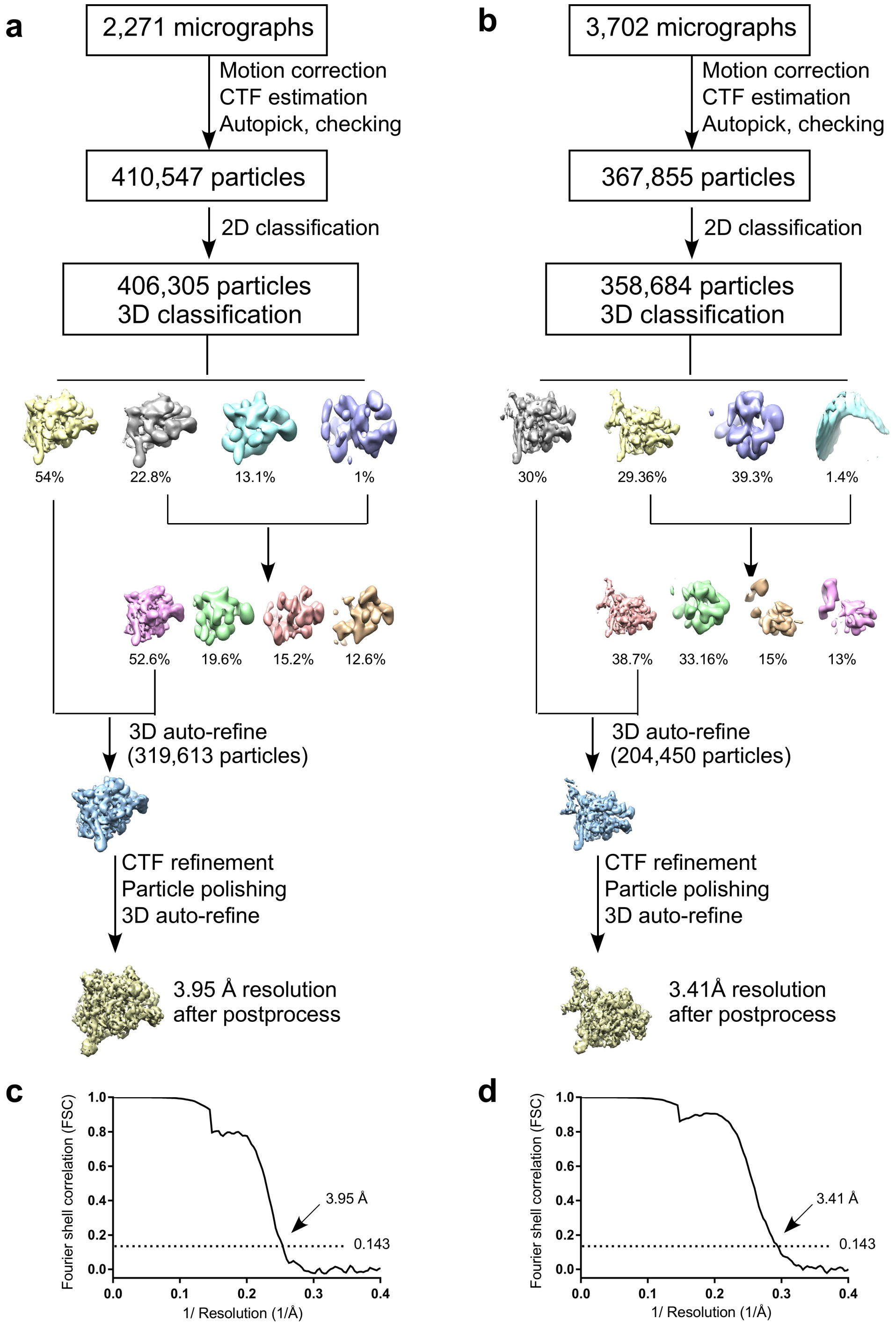

Supplementary Figure 3. The processing pipelines for cryo-EM map construction. Related to Figure 1. The flowchart of image processing for cryo-EM data of **(a)** *Xoo* P7-TEC and **(b)** *Xoo* P7-NusA-TEC**.** The numbers of micrographs collected for each dataset and single particles used in each step are listed. **(c)** The Gold-standard FSC of *Xoo* P7-TEC dataset. The dotted line represents the 0.143 FSC cutoff, which indicates a nominal resolution of 3.95 Å.**(d)** The gold-standard FSC of *Xoo* P7-NusA-TEC dataset. The dotted line represents the 0.143 FSC cutoff, which indicates a nominal resolution of 3.41 Å.

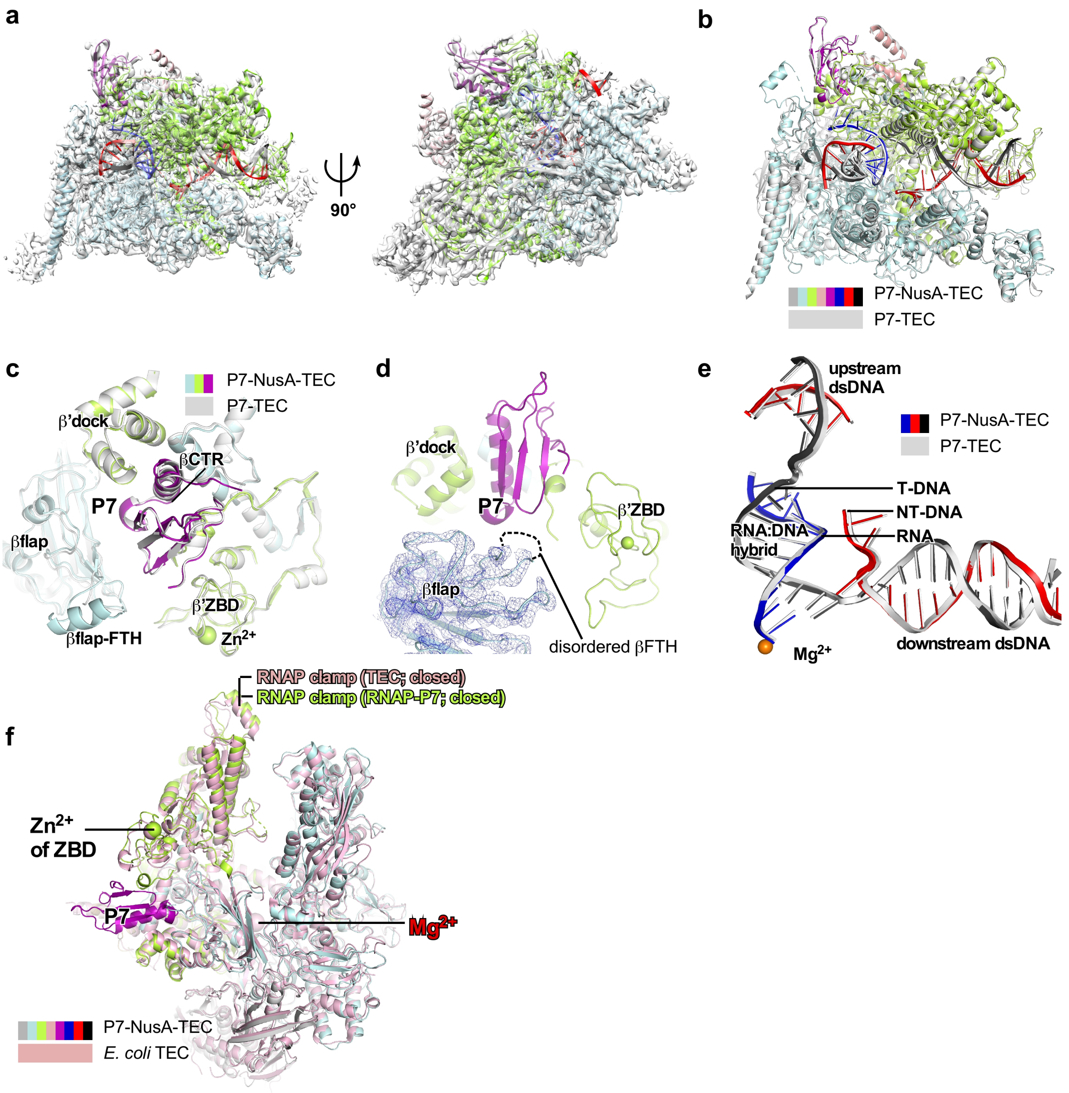

Supplementary Figure 4. The structural comparison of *Xoo* P7-TEC, *Xoo* P7-NusA-TEC, and *E. coli* TEC. Related to Figure 1. (a) The map and model of *Xoo* P7-TEC in two view orientations. The cryo-EM map is shown as gray surface. **(b)** Superimposition of overall structures of *Xoo* P7-TEC and *Xoo* P7-NusA-TEC. **(c)** Structural superimposition of P7 binding site in structures *Xoo* P7-TEC and P7-NusA-TEC. **(d)** Electron density map shows the RNAP- βFTH is disordered in the structure of P7-TEC. **(e)** Structural superimposition of nucleic-acid scaffolds of *Xoo* P7-TEC and P7-NusA-TEC. **(f)** Superimposition of overall structures of *Xoo* P7-NusA-TEC and *E. coli* elongation complex (PDB: 6ALF) [https://www.rcsb.org/structure/6ALF] showing that the RNAP-β' clamp of the two structures adopts the same closed-conformation.

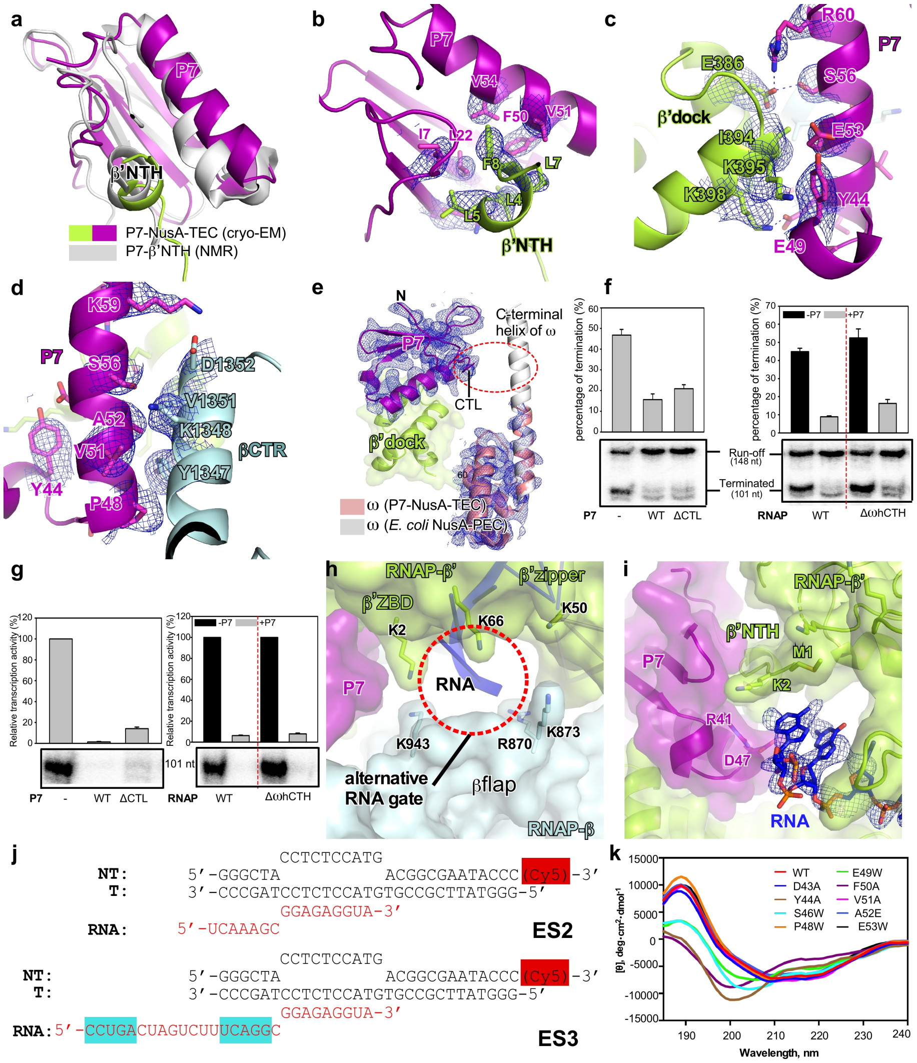

Supplementary Figure 5. P7 binds to the RNA-exit channel of RNAP. Related to Figure 2. (a) The superimposition of the cryo-EM structure of P7-NusA-TEC and the NMR structure of P7-β'NTH binary complex (PDB: 2MC6) [https://www.rcsb.org/structure/2MC6]. The cryo-EM electron density maps (blue mesh) for residues on **(b)** the interface of P7 and RNAP β'NTH**, (c)** the interface of P7 and RNAP β'dock, and **(d)** the interface of P7and RNAP βCTR. **(e)** The cryo-EM electron density map of P7 and RNAP-ω subunit in the structure of P7-NusA-TEC. The red dashed circle highlights possible interactions of the P7 C-terminal loop and the modeled extended C-terminal half helix of RNAP-ω subunit in the structure of P7-NusA-TEC. **(f)** Disrupting the proposed possible interface between P7 and RNAP-ω subunit (P7-ΔCTL, deleting the P7 C-terminal loop; ΔωhCTH, removing the ω C-terminal half helix) shows effect on the antitermination effect of P7. The area of terminated transcript contains two RNA bands, of which only the upper one is affected by P7 and thereby quantitated. **(g)** Minor effect on the transcription inhibition effect of P7. **(h)** The alternative RNA-exit gate in the structure of P7-NusA-TEC. **(i)** The RNA in the RNAP-active-center cleft was guided by residues of RNAP-β' subunit and P7 to the new exit gate. **(j)** The elongation nucleic-acid scaffolds used in Fig. 3j (ES3) and Fig. 3k(ES2). A Cy5 fluorophore was labeled at the 3'-end of the nontemplate DNA strand; the cyan box highlights the hairpin-forming sequence. (k) Circular dichroism spectra of wild-type or derivatives of P7 proteins. The source data of supplementary Fig. 5f-g and 5k are provided in the Source Data file.

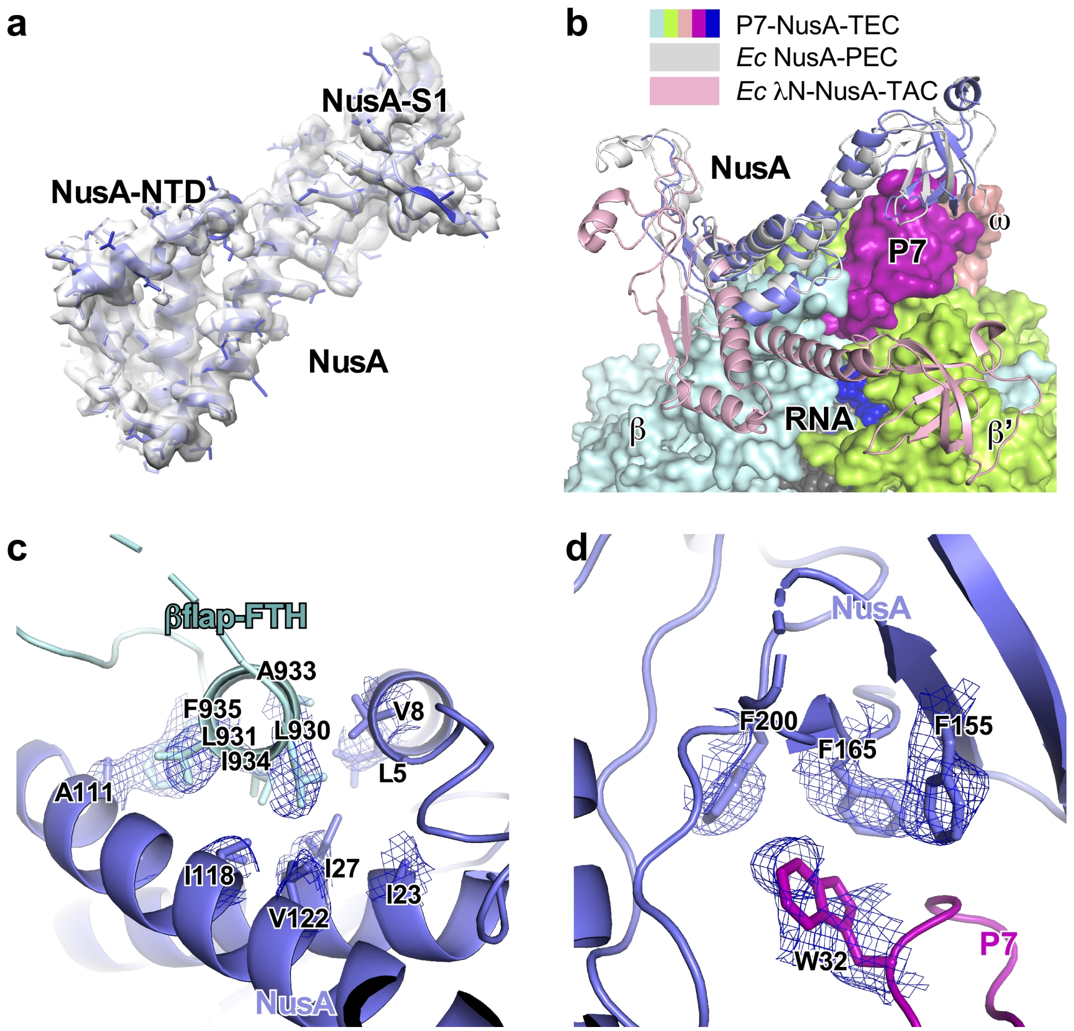

Supplementary Figure 6. NusA interacts with RNAP and P7. Related to Figure 4. (a) The cryo-EM map of NusA was shown as gray surface. **(b)** The superimposition of NusA in P7-NusA-TEC, *E. coli* NusA-PEC (PDB: 6FLQ) [https://www.rcsb.org/structure/6FLQ] and *E. coli* λN-NusA-TAC (PDB:6GOV) [https://www.rcsb.org/structure/6GOV]. **(c)** The detailed interactions between NusA and βFTH in the structure of P7-NusA-TEC. **(d)** The detailed interactions between NusA and P7. The cryo-EM electron density map for side-chains of amino acids is shown as mesh and colored in blue.

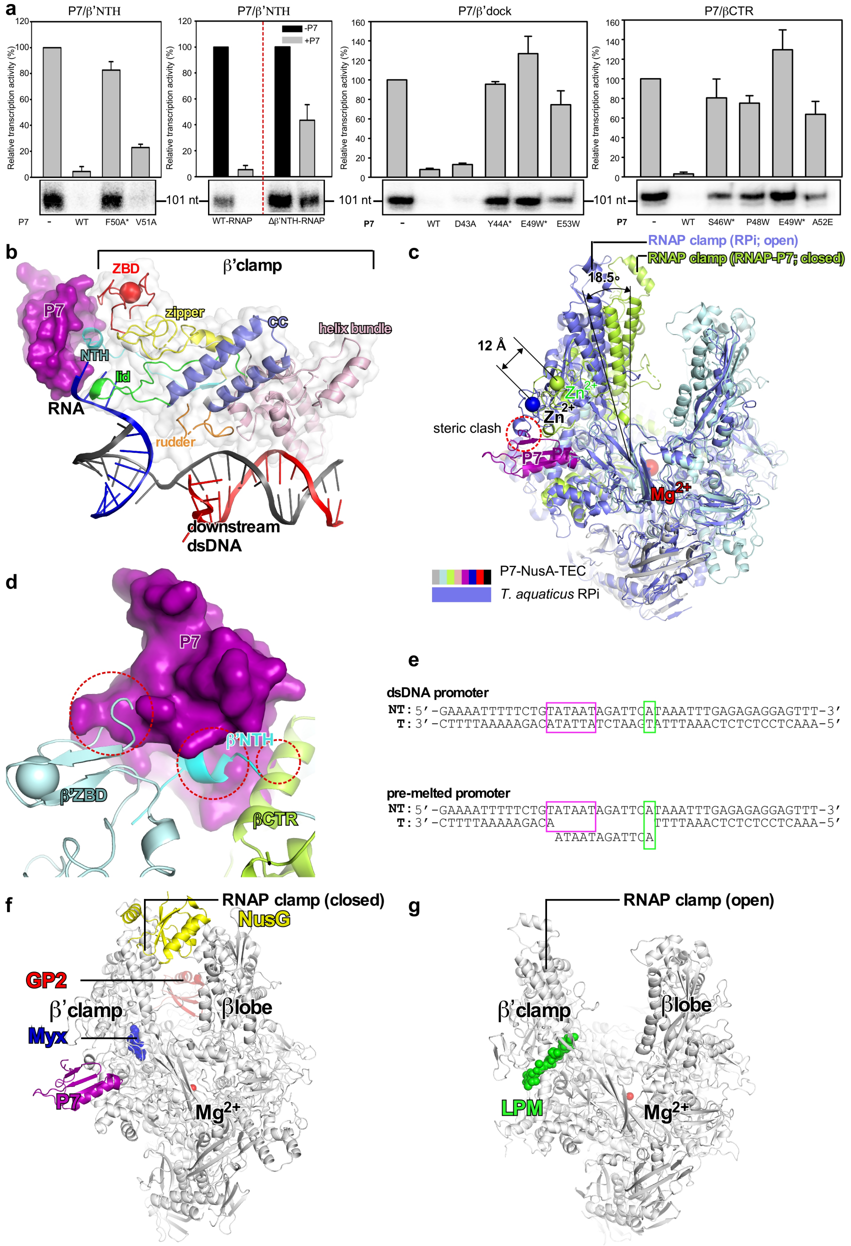

Supplementary Figure 7. P7 inhibits transcription initiation by jamming the RNAP clamp. Related to Figure 5. (a) Disrupting the interactions of P7/β'NTH, P7/β'dock, and P7/βCTR greatly alleviated inhibition of transcription initiation by P7. The experiments were repeated in triplicate, and the data were presented as mean ± S.E.M**. (b)** The RNAP β'clamp comprises β'NTH (residues 1-10), β'ZBD (residues 65-94), β'lid (residues 250-264), β'CC (residues 264-306), β'rudder (residues 306-327), and a helix-bundle (residues 112-238) near the dsDNA channel. **(c)** Structural superimposition of *T. aquaticus* RPi (PDB: 5TJG; open clamp) [https://www.rcsb.org/structure/5TJG] with P7-NusA-TEC (closed clamp) shows a 18° swinging of the clamp domain and a concomitant 12-Å movement of β'ZBD. **(d)** A close-up view of the steric clash between P7 and β'NTH/β'ZBD/βCTR in the open-clamp conformation of RNAP. The clash sites were highlighted with dashed red circles. **(e)** The promoter sequence used in the fluorescence polarization experiments in Fig. 5G and 5H. **(f)** The binding locations of RNAP inhibitors (Myxopyronin, Myx) or transcription factors (NusG and GP2) locking the RNAP clamp in the closed conformation. **(g)** The binding location of Lipiarmycin (LPM) locking RNAP in an open conformation. The source data of supplementary Fig. 7a are provided in the Source Data file. The asterisks in (a) indicate substitutions, which might also affect P7 overall structure .
