# Supplementary material for: Structural basis for transcription antitermination at bacterial intrinsic terminator
Source: Nat Commun. 2019 Jul 11;10:3048. doi: 10.1038/s41467-019-10955-x (PMC6624301; doi:10.1038/s41467-019-10955-x)
Supplement: Supplementary file 3 — Description of Additional Supplementary Files [file 41467_2019_10955_MOESM3_ESM.pdf]

Description of Additional Supplementary Files

File name: Supplementary Data 1

Description: The primer sequences in the study
